# Simultaneous Stress and Field Control of Sustainable Switching of Ferroelectric Phases

**DOI:** 10.1038/srep13770

**Published:** 2015-09-08

**Authors:** P. Finkel, M. Staruch, A. Amin, M. Ahart, S.E. Lofland

**Affiliations:** 1 US Naval research Laboratory, Washington DC, 20375; 2 Naval Undersea Warfare Center (NUWC), Newport, Rhode Island 02841, USA; 3 Geophysical Laboratory, Carnegie Institution of Washington, Washington DC 20015, USA; 4 Department of Physics and Astronomy, Rowan University, Glassboro, New Jersey 08028, USA

## Abstract

In ferroelectrics, manifestation of a strong electromechanical coupling is attributed to both engineered domain morphology and phase transformations. However, realization of large sustainable and reversible strains and polarization rotation has been limited by fatigue, nonlinearity and hysteresis losses. Here, we demonstrate that large strain and polarization rotation can be generated for over 40 × 10^6^ cycles with little fatigue by realization of a reversible ferroelectric-ferroelectric phase transition in [011] cut Pb(In_1/2_Nb_1/2_)O_3_-Pb(Mg_1/3_Nb_2/3_)O_3_-PbTiO_3_ (PIN-PMN-PT) relaxor ferroelectric single crystal. Direct tuning of this effect through combination of stress and applied electric field, confirmed both macroscopically and microscopically with x-ray and Raman scattering, reveals the local symmetry while sweeping through the transition with a low applied electric field (<0.2 MV/m) under mechanical stress. The observed change in local symmetry as determined by x-ray scattering confirms a proposed polarization rotation mechanism corresponding to a transition between rhombohedral and orthorhombic phases. These results shed more light onto the nature of this reversible transformation between two ferroelectric phases and advance towards the development of a wide range of ferroic and multiferroic devices.

Ferroic materials with extraordinary enhanced response of order parameters (magnetization, polarization, and strain) to external physical stimuli are of significant interest with both fundamental and technological importance. Effective energy conversion between elastic and electric fields through mutual control of corresponding stress and polarization is crucial for piezoelectric actuators, transducers, and low-power sensors^1^,^2^,^3^. Recently, the discovery of large voltage tuning of magnetism in multiferroic magnetoelectric heterostructures has further motivated research on complete and reversible control of electrically driven strain^4^,^5^. Noteworthy, materials with compositions close to a morphotropic phase boundary (MPB) region separating tetragonal and rhombohedral phases have demonstrated enhanced coupling in both ferroelectric and ferromagnetic materials, establishing the universal role of the MPB^6^,^7^. For electromechanical conversion, the breakthrough of large piezoelectric response in relaxor ferroelectric crystals near MPB has been attracting tremendous attention for over a decade with maximum achievable strain more than 1%, almost an order of magnitude enhancement compared to conventional ceramics^8^,^9^,^10^,^11^. Multiple studies have concluded that electromechanical coupling in relaxor piezo-crystals can be maximized by arranging special domain configurations in systems with adaptive domain morphoplogy^9^,^12^,^13^. Yet there are several primary challenges that have limited the operation of relaxors including strong nonlinearity and hysteresis^14^, and thus the realization of large sustainable and reversible strains has remained elusive.

However, recently it was demonstrated that a stress biased [0 1 1] cut relaxor ferroelectric Pb(In_1/2_Nb_1/2_)O_3_-Pb(Mg_1/3_Nb_2/3_)O_3_-PbTiO_3_ (PIN-PMN-PT) single crystal can generate reversible strain >0.35% at remarkably low field of order 0.1 MV/m^15^. This behavior has been attributed to an inter-ferroelectric phase transition marked by a sharp jump in strain. The mechanism responsible for this transformation was similar to that identified earlier by Viehland^16^ for [1 0 0] crystals. Poled [0 1 1] crystals are in a stable multidomain state in with only two variants of the rhombohedral (R) structure (space group *R*3*m*) with polarization aligned along the cubic [1 1 1] and [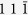] directions. Under the collective effect of a critical stress σ_*c*_ and critical electric field *E*_*c*_, the polarization rotated to the cubic [0 1 1] direction and it was hypothesized that there was an accompanying phase transformation from R symmetry to a monodomain orthorhombic (O) phase consistent with Devonshire theory for this proposed polarization rotation mechanism^15^. In principle, this picture is consitent with an earlier assertion that MPB can be moved by either external field, stress, or their combination^17^,^18^. By manipulating the MPB in this manner, this large nonlinear strain response can be repeatably harnessed and in fact with hysteresis much lower than for 180^o^ polarization switching.

The reversibility of the stress-strain curve has been previously confirmed on a microscopic level with *in-situ* x-ray diffraction analysis^19^. However that study did not investigate simultaneous tuning of the transition under both stress and electric fields nor did it establish unequivocal evidence of a change in the local symmetry of the phases. The ferroelectric phase diagram near the MPB^20^,^21^ is quite complex and unambiguous identification of the symmetry under various boundary conditions will help to resolve the complicated physics of these materials. In this work, we report on direct observation of reversible phase transitions as a function of electric field and stress in near-MPB [0 1 1] poled PIN-PMN-PT single crystals (PT ~ 29%), with geometry of the sample and applied fields shown in Fig. 1.

## Results

The stress-strain response of the crystal typically (Figure S1) exhibits a very prominent and abrupt hysteretic elastic response and shows that positive dc bias leads to destabilizing of the R state. Increasing electric field leads to a reduction in the critical stress which appears to destabilize the R state. The inset of Figure S1 shows the interdependence of the critical stress σ_*c*_ and critical electric field *E*_*c*_.

To investigate possible structural transitions, polarized (VV) and de-polarized (VH) Raman scattering measurements were done as a function of electric field *E* at a stress of 20 MPa with an Acton SP300i spectrometer with a 532 nm laser source with the average incident power of <10 mW. Spectra were taken at 298 K with an acquisition time of 100 seconds. The observed modes are all consistent with previous work on relaxor ferroelectrics^22^,^23^. With applied electric field cycled ± 0.2 MV/m, there is a noticeable change in susceptibility (Fig. 2) and the intensities of the peak centered at 54 cm^−1^ (VH polarization) shows an asymmetric hysteresis loop, suggestive of a structural transition. However, no shift in peak position or abrupt discontinuity in intensity ratios are observed in contrast to what has previously been found at *T*_*C*_ or at the tetragonal-to-rhombohedral transitions^22^,^23^.

**Figure 1:**
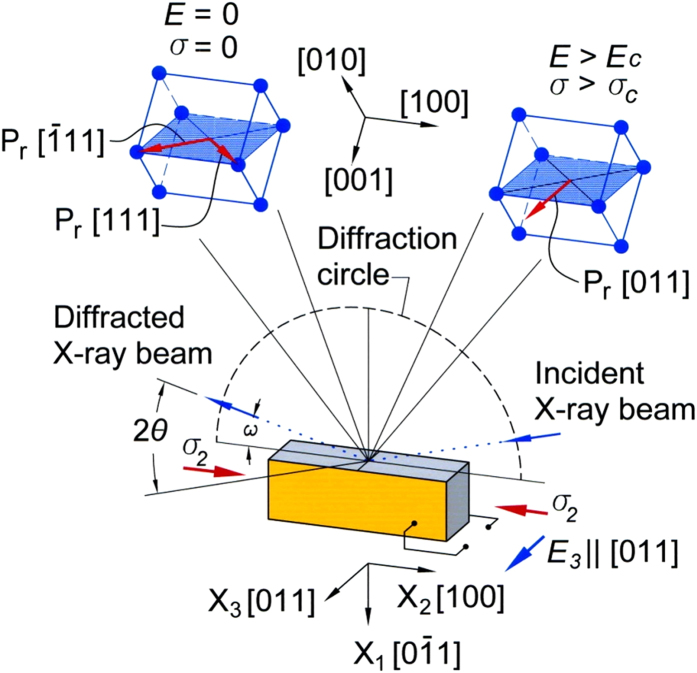
Polarization rotation due to ferroelectric –ferroelectric rhombohedral (R) to orthorhombic (O) phase transformation in domain engineered ferroic crystal (a) Schematic of the formation of two polarization variants (*P*_[111]_ and (*P*_[−111]_) and their switching from polydomain to monodomain under the application of a local electric field E (along [011]) and/or uniaxial compressive stresses σ (along [100]) above critical values Ec and σc. Reversible polarization switching results from the charged domain walls^17^.

**Figure 2:**
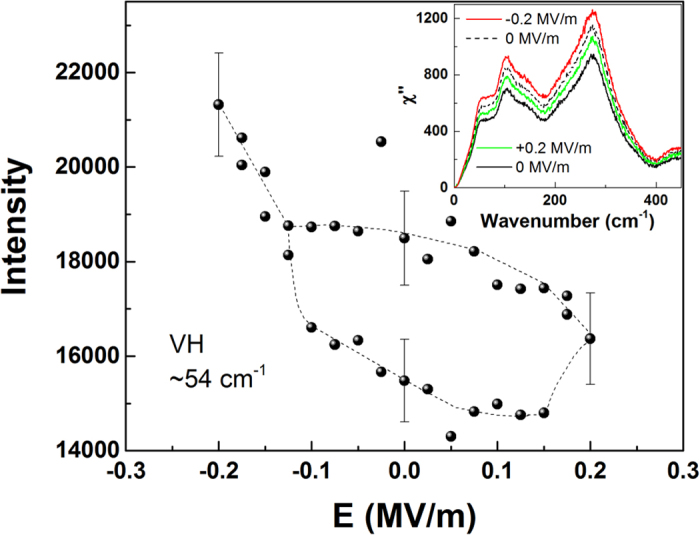
Electric field dependent integrated intensity of peak at 54 cm^−1^ in VH mode for a pre-stressed PIN-PMN-PT single crystal. Dashed lines are given as a guide for the eyes. Error bars are shown for select points confirming that the hysteresis is statistically significant. The inset shows the change in susceptibility at several values of electric field.

To understand the evolution of the local crystal structure as functions of σ and *E*, x-ray diffraction experiments were conducted on a crystal placed in a custom-built loading fixture with stress applied along the *x*_2_ axis of the crystal and electrodes were attached to the *x*_3_ faces (Fig. 1). The goniometer of the x-ray diffractometer was then aligned to the *x*_1_ plane. Measurements were taken in the Bragg-Brentano geometry in the *x*_2_ plane as functions of stress and electric field. Reciprocal space maps (RSM) were taken of the cubic (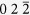), (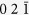), and (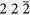) planes.

**Figure 3:**
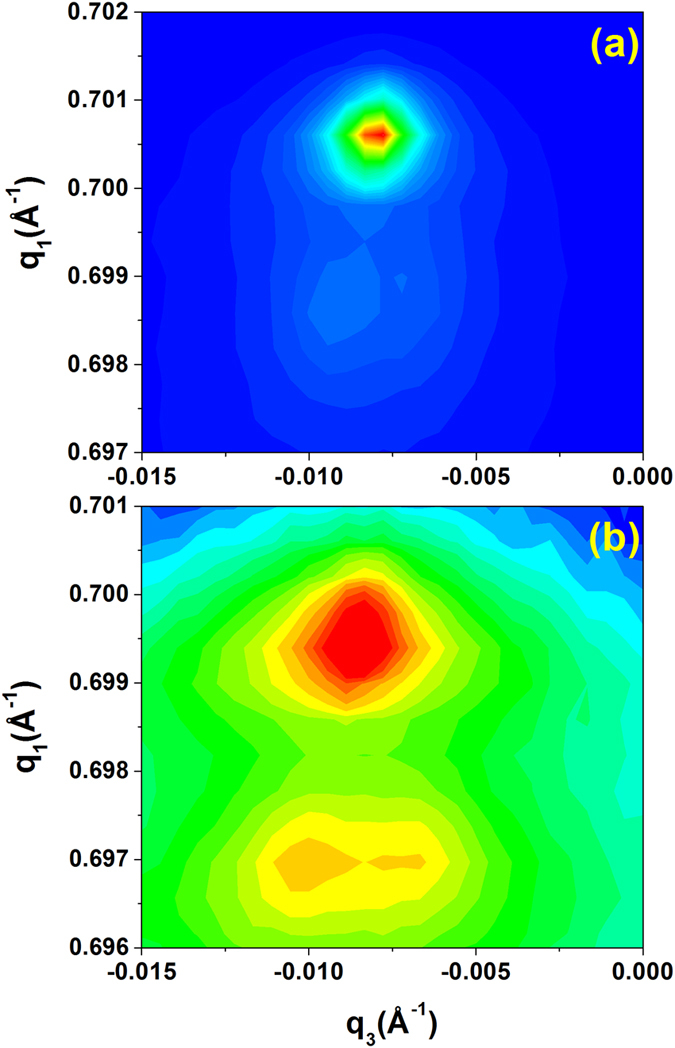
Logarithmic representation of the reciprocal space maps of the cubic (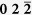) reflection (a) at 0 MPa and (b) at 20 MPa. In (**a**), the sample is predominately rhombohedral and there are only two clear reflections, the main (220) reflection and the minor (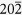) one. In (**b**), the sample is in the orthorhombic state with the (040) the main reflection along with (400) reflection being observed. The small second peak indicates the presence of weak twinning. The fact that q_3_ is not zero is indicative of a small miscut in the sample.

RSMs taken at σ = 0 and *E* = 0 displayed one dominant peak accompanied by one or more minor peaks, indicating the presence of some twinning (Fig. 3a). While a fully twinned R crystal has 8 degenerate variants, poling along the *x*_3_ axis breaks the symmetry such that ideally only two variants along [1,1,1] and [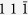] (Fig. 1) should exist in the present crystal, and those two variants should be indistinguishable in this set of RSMs. Thus, these minor peaks suggest a small fraction of the variants with polarization in the *x*_1_-*x*_2_ plane of the sample. These could be due to insufficient field during poling to completely polarize the sample or a slight miscut in the sample surface. Nonetheless, below σ_*c*_ and *E*_*c*_, as expected, the RSMs were found to be consistent with that of R structure (Table 1).

On the other hand, RSMs measured above σ_*c*_ and corresponding *E*_*c*_ (Fig. 3b) also showed multiple peaks but the resulting *d* spacings were incompatible with R structure. Since BaTiO_3_ is a prototypical perovskite ferroelectric and is isostructural to the R phase of the present crystal, we considered its other ferroelectric space groups as candidates for that of PIN-PMN-PT, namely, tetragonal *P*4*mm* and orthorhombic *Amm*2. Table SI lists the Miller indices of the allowed reflections of the aforementioned phases of BaTiO_3_^24^ and their relationship to those of the cubic phase. Since the degeneracy of the reflections for cubic (1 1 1) is not broken by tetragonal symmetry yet two peaks were observed in the corresponding RSM, the only viable candidate is O symmetry.

Within the resolution of the measurements, all the RSMs were compatible with a twinned orthorhombic crystal (Table SI). The main peak found in the cubic (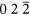) RSM (Fig. 3b) is identified as the (0 4 0) reflection, confirming that the 2 principle variants from the R state do collapse into one in the O state, with the ***a*** axis of the crystal, the polarization direction, along *x*_3_.

**Figure 4:**
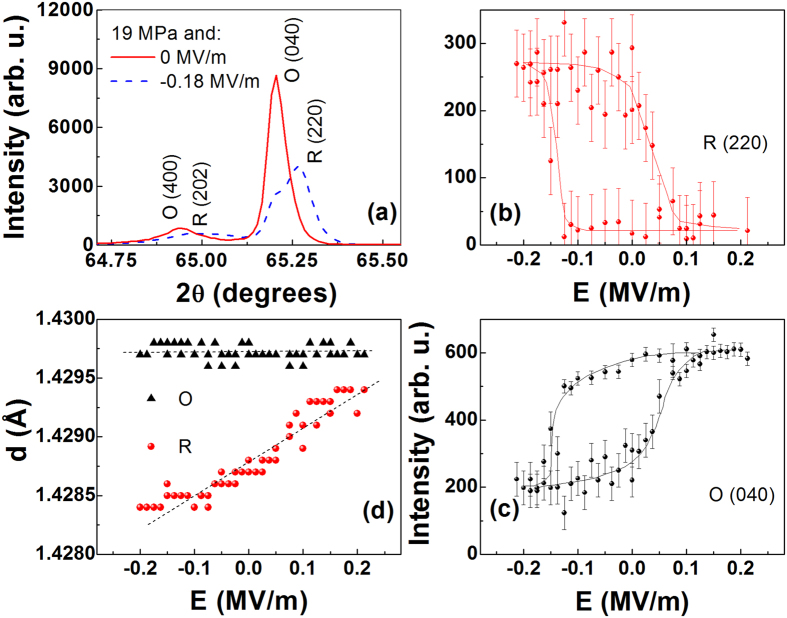
(**a**) The xrd pattern at 19 MPa at zero field (red) and at −0.18 MV/m (blue). Note the presence of the remanent of the orthorhombic (0 4 0) peak at E = −0.18 MV/m. Integrated intensity of (**b**) rhombohedral (220) and (**c**) orthorhombic (040) peaks as functions of electric field at 19 MPa. The lines serve as guides to the eye. (**d**) Electric field dependence of the lattice spacing of the R (2 2 0) and O (0 4 0) at 19 MPa. Both phases act as simple piezoelectrics with linear strain dependence.

Near 19 MPa *E*_*c*_ is near zero (Figure S1), and the phase is O for positive E > 0 or R for E < 0. Figure 4(a) shows the results of conventional Bragg-Brentano measurements of the cubic (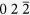) peak at 19 MPa. Figure 4(b,c) show the intensity of the principle peaks of the two phases as *E* is cycled ± 0.2 MV/m, clearly demonstrating reversibility. Remarkably *E* field cycling exhibits much lower hysteresis as compared to 180^o^ polarization switching with coercive field (*E*_*C*_) ~ 0.67 MV/m. Figure 4(d) shows the *E* dependence of the *d* spacing of the cubic (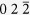) reflections [i.e. O (0 4 0) and R (2 2 0)]. It is notable that the lattice parameters of both phases are linearly dependent on field, as though each phase were behaving as simple piezoelectrics. No other metastable intermediate phases were observed between the two stable states while cycling electrically through the transition.

## Discussion

**Figure 5:**
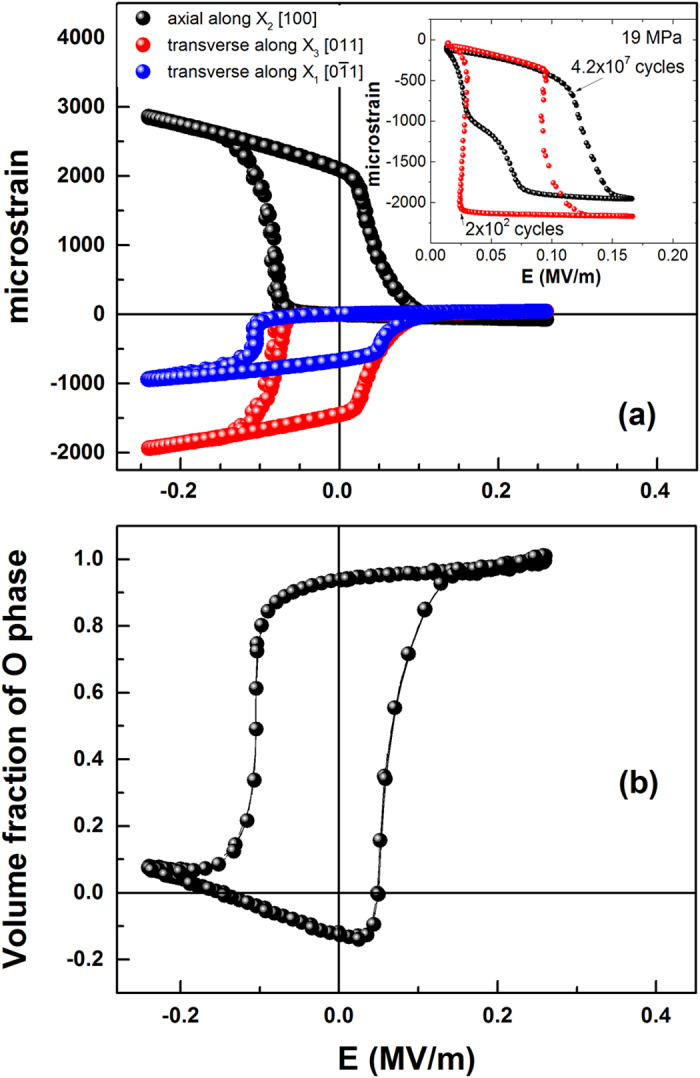
(**a**) Measured bulk strain at 21 MPa as a function of the electric field. Insert shows the electrically driven strain at 19 MPa at different cycles. It should be noted that electric field bias needed to induce transition varies with applied pre-stress. (**b**) Volume fraction of the orthorhombic phase based on the results of Fig. 4(**b**,**c**). Note that there are no free parameters; i.e. this is not a fit.

One way to compare the bulk strain ε (Fig. 5a) to the x-ray results is to express the average *d* spacing 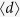 as


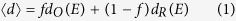


where *d*_*O*_ and *d*_*R*_ are the measured lattice spacings of the O and R phase, respectively, and *f* the volume fraction of the O phase. Thus,


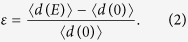


Combining Equations (1) and (2) allows one to deduce *f*(*E*) for the O phase.


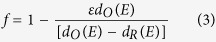


The resulting graph (Fig. 5b), shows that the bulk measurements confirm that the entire sample transforms between the O and R phases. These results are qualitatively similar to the x-ray intensities of Fig. 4, but a direct comparison is not possible due to the fact that a complete structural refinement is necessary at each data point for quantitative analysis of the intensity.

The phase transition is shown to be R-O, without presence of an intermediate monoclinic M_B_ phase. Thus, one can conclude that the previously reported M_B_ phase in fact a phase whose local symmetry is either R or O or a combination of both. As given by equation (1), the changes in lattice parameters are described by changes in the volume fraction of the O and R phases, as one grows from the other. In some way, the data give support to the adaptive phase theory^25^. Following this theory, the local symmetry is either R or O along the [011]. With applied ordering fields (stress or electric), the R nano-twins would change their distribution with increasing field, which is in line with the present x-ray results.

To describe the boundary between the O and R phases, let us consider the Gibbs free energy *G*:


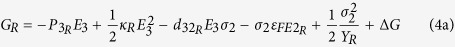



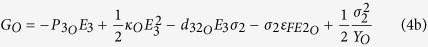


where *P* is the spontaneous polarization, *d*_32_ the piezoelectric coefficient, κ the dielectric constant, ε_*FE*_ the spontaneous ferroelectric strain, *Y* Young’s modulus, and the *R* and *O* subscripts correspond to the R and O phases, respectively. The Δ*G* term in the *G*_*R*_ is due to the difference in energies between R and O phases at zero field and stress related to terms due to dielectric stiffness tensors. Accepted values for the material characteristics are 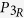 = 0.26 C m^−2^, κ_*R*_ = 4500, 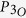 = 0.32 C m^−2^, κ_*O*_ = 700^26^,^27^,^28^, and the present x-ray and stress-strain measurements yield 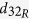 = −1.2 × 10^−9^ m V^−1^, 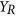 = 20 GPa, 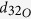 = −0.2 × 10^−9^ m V^−1^, 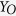 = 70 GPa, and 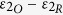 = −0.0035. A rather small 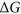 value of −70 kJ m^−3^ reproduces the phase boundary shown in Figure S1(a). The latent heat Δ*Q* was calculated from the entropy change Δ*S* via the Clausius equation from the values from Figure S1(b):


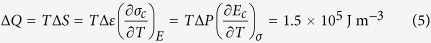


This value is quite close to that predicted by first principle calculations^29^,^30^ Relatively small value for Δ*Q* allows the phases readily to transform with small hysteresis. The low energy between R and O phases allows practically simultaneous polarization switching which occurs at fields much lower than the coercive field for the R phase.

The observed lack of fatigue may be related to the sharpness of the transition. While the bulk strains may be large, the coherent switching of the phases means that there is no internal strain in the crystal due to the two structures. In addition, this is likely accompanied by low domain wall energies in twinned crystal domains and is in accord to adaptive phase model^21^.

In general, application of either stress along **x**_**2**_ or electric field along **x**_**3**_ leads to slight monoclinic distortion of the R structure, but the distortion is so small that it cannot be resolved by the present x-ray measurements. However, since the monoclinic phase is thought to be unstable^31^, any sizable stress or field causes the crystal to transform to orthorhombic symmetry. Previous reports of monoclinic phases observed by x-ray and neutron studies may be due to a mixture of R and O phases. It is evident that the precise mapping of phase boundaries is still deceptive due to extreme sensitivity of all phases to external physical parameters and even to history of poling conditions. A complete understanding of this ferroelectric – ferroelectric transition is expected to further development of a wide range of devices. We envision that these results could have an impact not only for novel transducers but also for laminated multiferroics where the large transitional strain can give rise to giant magnetoelectric coupling^32^,^33^,^34^,^35^.

In summary, we have shown that domain engineered [0 1 1] crystal of PIN-PMN-PT transforms between R and O phases by electric field, mechanical stress, or their combination. The crystal can undergo a large number of cycles without fatigue due to coherent switching related to the small energy differences in the two states. Most importantly, the present results from both macroscopic and microstructural studies shed more light onto nature of this reversible transformation between two ferroelctric phases and the suggested metastability of the monoclinic phase, thus closing the gap in the understanding of these materials.

## Methods

### Measurement of elastic properties

The PIN-PMN-PT samples used in this investigation provided by HC materials were sliced into 4 × 4 × 12 mm^3^ bars and cut and poled along [0 1 1] direction. Isothermal compression-decompression experiments along [0,0,1] direction were conducted using approximately 0.07 Hz half sine wave pressure cycle between 0 to approximately 50 MPa at each preset dc bias electric field. The experimental details have previously been described elsewhere^36^.

### Raman spectroscopy measurements

Acton SP300i spectrometer with a 532 nm laser source with the average incident power of <10 mW was used in this study and measurements performed at room temperature.

*X-ray measurements*: X-ray studies were done with a Panalytical Empyrean diffractometer with a Pixcel two-dimensional detector. Measurements were done at room temperature with Cu K_α1_ radiation with a Ge two-bounce monochromator with a 1/32° divergence slit and a 2 mm beam mask.

## Additional Information

**How to cite this article**: Finkel, P. *et al.* Simultaneous Stress and Field Control of Sustainable Switching of Ferroelectric Phases. *Sci. Rep.* **5**, 13770; doi: 10.1038/srep13770 (2015).

## Supplementary Material

Supplementary Information

## Figures and Tables

**Table 1:** Structure and lattice parameters at *E* = 0 as calculated from RSMs.

**Stress (MPa)**	**Structure**	**a (Å)**	α **(degrees)**	
0	Rhombohedral	4.0416 (7)	89.87 (3)	
19	Rhombohedral	4.0441 (5)	89.85 (2)	
		*a* (Å)	*b* (Å)	*c* (Å)
20	Orthorhombic	5.7496 (11)	5.71804 (3)	4.0176 (59)
