# Supplementary material for: Simultaneous Stress and Field Control of Sustainable Switching of Ferroelectric Phases
Source: Sci Rep. 2015 Sep 8;5:13770. doi: 10.1038/srep13770 (PMC4561888; doi:10.1038/srep13770)
Supplement: Supplementary Information [file srep13770-s1.pdf]

Simultaneous Stress and Field Control of Sustainable Switching of Ferroelectric Phases

By P. Finkel *, M. Staruch, A. Amin, M. Ahart, and S.E. Lofland

Supplemental Materials

Elastic response of the samples as a function of electric field and temperature was measured in isothermal compression-decompression experiments along [1 0 0] direction and experiments were conducted using approximately 0.07 Hz half sine wave pressure cycle between 0 to approximately 50 MPa at each preset dc bias electric field. The ferroelectric rhombohedral (F_R) – ferroelectric orthorhombic (F_O) transition at room temperature (shown in Figure S1(a)) is remarkably sharp with a discontinuity in the stress-strain curve near ~21 MPa for 32-mode samples. Domain engineered PIN-PMN-PT single crystals exhibit four F_R and two F_R domains for (0 0 1) and (0 1 1) cut & poled single crystals, respectively. F_R rhombohedral multi-states were stabilized by DC bias field along [0 0 1] poling direction while F_O orthorhombic single domain was stabilized by DC bias field along [0 1 1] poling direction. The stabilization of the F_O phase in 32-cut and [0 1 1]-poled single crystal could be explained by reorientation of the polarization vector P_s from <1 1 1> directions to <0 1 1> directions. An applied bias electric field or higher temperature (up to 50 ^{o}C, not shown) shift this transition to lower critical stresses. At high bias field > 1 MV/m, the crystal is totally transitioned to the O state and the stress-strain responses becomes linear with flat slope.

Stress-field-temperature 3D stability diagram can be established defining the linear regions based on critical values of stress, electric bias and temperature from the stress-strain measurements and is shown in Figure S1(b). Under certain boundary conditions defined by critical compressive stress and applied bias field (deduced from the quasistatic elastic isothermal response, inset of Figure S1(a)) this stability diagram depicts the states on the surface representing the R-O-R boundary as a function of temperature. Color mapping establishes the critical stress versus critical electric field relationship for specific corresponding temperatures ranges. Thus the F_O state is stable for any stress, field, and temperature above this surface, while F_R is stable below this surface.

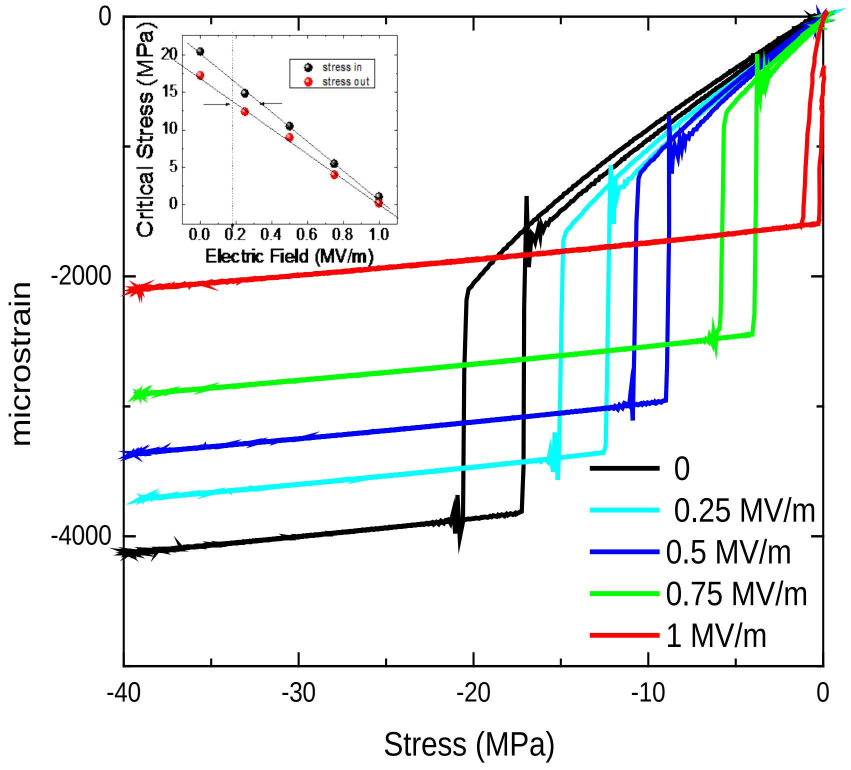

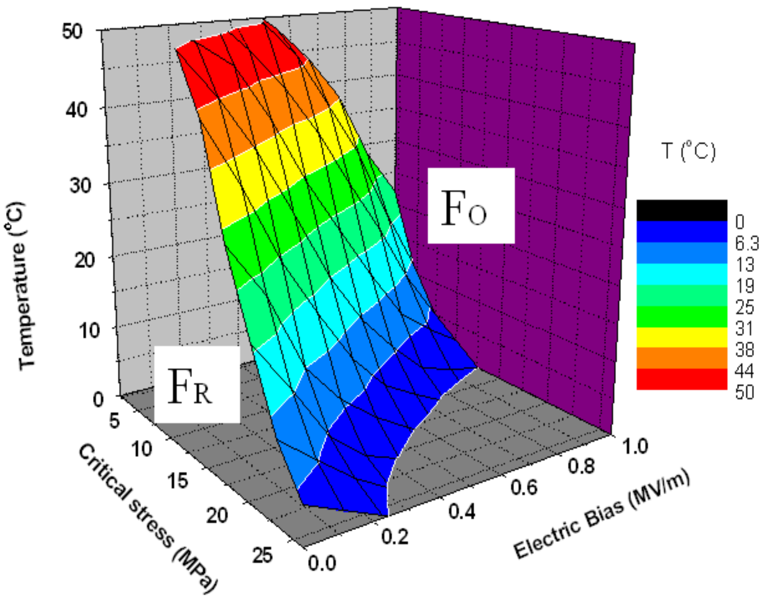

Figure S1. (a) Room temperature elastic response at various electric field bias. The inset shows the relationship between the critical electric field and critical stress. (b) Stress-field-temperature stability surface for [011] poled PIN-PMN-PT samples, where F_R and F_O represent stable ferroelectric rhombohedral and ferroelectric orthrhombic states, respectively.

Table SI

Miller indices of cubic space group and relationship to reflections of related space groups of lower symmetry.

Cubic<br>Pm3m	Rhombohedral<br>R3m	Tetragonal<br>P4mm	Orthorhombic<br>Amm2
(2 1 0)	(2 1 0)<br>$(2\ 0\ \bar{1})$	(0 1 2)<br>(1 2 0)<br>(0 2 1)	(0 1 3)<br>(0 3 1)<br>(1 2 2)<br>(2 1 1)
(1 1 0)	(1 1 0)<br>$(1\ 0\ \bar{1})$	(1 1 0)<br>(0 1 1)	(2 0 0)<br>(0 2 0)<br>(1 1 1)
(1 1 1)	(1 1 1)<br>$(1\ 1\ \bar{1})$	(1 1 1)	(1 0 2)<br>(1 2 0)
